# Active Packaging Based on a PET/PP Food-Grade Film Coated with Pullulan and Clove Essential Oil: Physicochemical and Antimicrobial Properties

**DOI:** 10.3390/molecules30102118

**Published:** 2025-05-10

**Authors:** Karolina Kraśniewska, Małgorzata Gniewosz

**Affiliations:** Department of Food Biotechnology and Microbiology, Institute of Food Sciences, Warsaw University of Life Sciences—SGGW (WULS—SGGW), Nowoursynowska Str., 159c, 02-776 Warsaw, Poland; malgorzata_gniewosz@sggw.edu.pl

**Keywords:** pullulan, clove essential oil, physical properties, antimicrobial and antioxidant activity, active packaging, spinach leaves

## Abstract

The objective of this study was to design an active packaging material based on a polyethylene terephthalate (PET)/polypropylene (PP) film modified with a pullulan coating enriched with 1, 5, and 10% of clove essential oil (CEO). The physical properties of modified PET/PP films, including opacity, UV, and light visible barrier properties, were evaluated, and calorimetric measurement of color (L*a*b*) was performed, followed by determination of their potential of antioxidant activity and antimicrobial properties against foodborne pathogenic bacteria (*E. coli*, *S.* Enteritidis, *S. aureus* and *L. monocytogenes*) were characterized. Additionally, the effectiveness of the active packaging in reducing pathogenic bacteria on spinach leaves was evaluated. The PET/PP film with a pullulan coating enriched with CEO did not affect their transparency. The incorporation of CEO improved the film’s UV and visible light barrier properties without causing noticeable color changes while also exhibiting good antioxidant and antimicrobial activity. Furthermore, the application of active packaging effectively inhibited the growth of pathogenic bacteria on spinach leaves, demonstrating its potential for food preservation.

## 1. Introduction

Packaging plays a crucial role in food preservation, acting as a barrier against physical, chemical, and microbiological factors. While conventional packaging is effective, it primarily serves as a passive protective barrier, limiting exposure to external influences. In recent years, significant advancements in packaging technology have introduced active packaging systems, which not only protect food but also interact with it to extend shelf life and enhance microbial safety [1,2]. Active packaging incorporates functional substances that interact with the food or its surrounding environment to maintain quality and freshness. This approach involves the controlled release or absorption of specific compounds. Based on their function, active packaging systems can be classified into active scavenging systems (absorbers) and active-releasing systems (emitters). Absorbers remove undesirable substances such as moisture, carbon dioxide, oxygen, and ethylene, while emitters introduce beneficial substances such as antimicrobial agents, carbon dioxide, antioxidants, and flavors [3,4].

Antimicrobial packaging is a type of active packaging that reduces, inhibits, or retards the growth of microorganisms that may be present in packed food [5,6]. Packaging with antimicrobial properties can be obtained by adding sachets or pads containing antimicrobial substances to the packaging [7,8,9], directly incorporating the antimicrobial agent into a polymer matrix [10,11] and applying an antimicrobial compound onto the polymer surface [12,13]. So far, various antimicrobial agents, including synthetic/chemical preservatives as well as natural agents, have been incorporated into packaging systems [14,15,16,17]. Nevertheless, consumer pressure to use natural agents for food preservation increased the interest in the use of naturally derived additives such as essential oils (EOs) [18]. EOs are complex mixtures of bioactive compounds derived from plant secondary metabolism. These volatile hydrophobic substances are primarily composed of terpenes, terpenoids, and phenylpropene compounds, which contribute to their diverse biological activities [19]. Clove (*Syzygium aromaticum*) is one of the most popular spices widely used as a natural food flavoring agent. Also, clove essential oil (CEO) obtained from the bud or leaves of the clove tree is considered a natural flavoring agent in various products, such as candies, baked foods, and beverages [20,21]. Furthermore, due to its excellent antimicrobial and antioxidant properties, it can also be used as a natural preservative [22,23]. Generally, CEO is composed of fractions consisting of bioactive compounds, mainly from the phenylpropanoid and sesquiterpene classes. A major compound found in both the bud and leaf clove oil is eugenol (a phenylpropanoid), followed by eugenol acetate, β-caryophyllene, and humulene [24].

Polyethylene terephthalate (PET)/polypropylene (PP) is widely used in the food packaging industry due to its affordability, transparency, and excellent thermal stability. The multilayer PET/PP film, compared to its monolayer counterpart, combines the mechanical strength and barrier properties of PET with the flexibility and heat-sealing ability of PP. As a result, this enhanced structure of multilayer PET/PP film exhibits better mechanical and barrier properties, and a heat-sealing ability which improves the stability and functionality of the final food packaging. This makes PET/PP film more effective for extending shelf life and maintaining food quality. Moreover, these two polymers are among the most commonly used ones for the development of active packaging [25,26]. Furthermore, bioactive compounds can be incorporated into the polymer to enhance its functionality to provide antimicrobial and antioxidant effects. However, the direct incorporation of active substances (e.g., essential oils) into polymeric matrices through the most commonly used techniques, such as extrusion, often results in the volatilization or thermal degradation of bioactive compounds due to high temperatures used during the process. Therefore, scientific studies already focus on using biopolymers with incorporated active substances as an additional layer to cover synthetic polymers [26,27,28,29].

Pullulan is an extracellular polysaccharide produced by *Aureobasidium pullulan* [30,31]. This biopolymer has good film-forming properties, as well as adhesive properties, which favor its application in films and coatings. Pullulan films are tasteless, odorless, and edible, while also exhibiting good optical properties, including high transparency and colorlessness [32,33]. Several studies have demonstrated the potential of pullulan, either alone or enriched with bioactive compounds, as a promising packaging material for coating food products [33,34,35,36,37,38]. In general, adding essential oils enhances the functional properties of pullulan coatings, such as their antimicrobial and antioxidant activity [34,38]. Moreover, studies have shown that incorporating clove essential oil into pullulan–gelatin and chitosan–pullulan films improves their mechanical strength, water resistance, barrier properties, and UV-blocking capabilities. These enhancements make pullulan-based films a highly functional and efficient solution for active food packaging [39,40].

Minimally processed vegetables are obtained using mild and non-thermal treatments, allowing them to retain their natural appearance, texture, and nutritional value. As a result, these products are highly perishable and vulnerable to post-harvest physiological changes and microbial contamination during transportation, handling, processing, storage, and distribution. Furthermore, the absence of thermal processing raises significant food safety concerns due to the potential presence of pathogenic microorganisms [41]. Among the various pathogens implicated in the contamination of minimally processed vegetables, *Escherichia coli* O157:H7, *Salmonella* spp., and *Listeria monocytogenes* have been the most frequently identified in previous studies [42,43]. Therefore, ensuring the microbiological safety of minimally processed produce while maintaining its sensory and nutritional quality over extended periods remains a major challenge in the food industry. To enhance the microbial safety and shelf life of minimally processed vegetables, active packaging systems with antimicrobial properties have been proposed as an innovative solution. Kwon et al. [43] investigated the use of polyvinyl alcohol (PVA) films incorporated with oregano essential oil and found that films containing 2–3% oregano oil effectively inhibited *Salmonella enterica*, molds, yeasts, and mesophilic aerobic bacteria on cherry tomatoes during storage. In other studies, Salmieri et al. [44] developed poly(lactic acid)–cellulose nanocrystal (PLA–CNC) films infused with oregano essential oil, which exhibited strong antibacterial activity against *Listeria monocytogenes* in mixed vegetables. PET/PP loaded with methyl gallate showed antioxidant activity and potential application in the preservation of perishable food such as lettuce and tangerine [29].

In this context, the present study aims to develop an innovative active packaging system based on a PET/PP film coated with pullulan enriched with clove essential oil. The main objective is to evaluate its physicochemical and antimicrobial properties, as well as its application in preserving minimally processed vegetables such as spinach. This study contributes to the search for sustainable and effective alternatives for preserving perishable foods.

## 2. Result and Discussion

### 2.1. Physical Characteristics of Films

The physical properties of unmodified and modified PET/PP films, including thickness, opacity, and color, are presented in Table 1. The results demonstrate that PET/PP films coated with pullulan exhibited greater thickness compared to uncoated/unmodified PET/PP films. Furthermore, the incorporation of essential oils into the pullulan coating increased the solids content of the coating, which, in turn, contributed to a further increase in thickness. A similar phenomenon was reported by Chu et al. [45], who also noted that the thickness of pullulan coatings was amplified with increasing concentrations of cinnamon essential oil in the biopolymer matrix.

**Figure 1:**
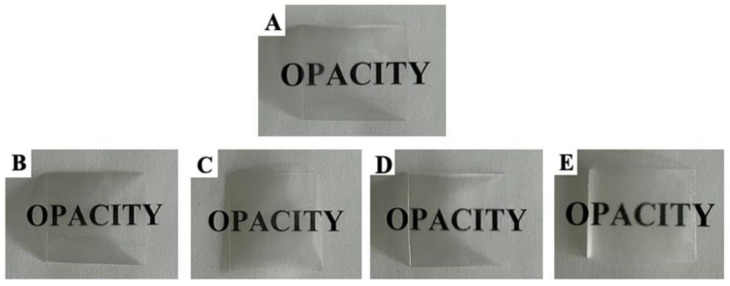
Visualization of film opacity: (**A**) PET/PP, (**B**) PET/PP/P, (**C**) PET/PP/P + 1%CEO, (**D**) PET/PP/P + 5%CEO, (**E**) PET/PP/P + 10%CEO. PET/PP—polyethylene terephthalate/polypropylene film, PET/PP/P—polyethylene terephthalate/polypropylene film with a layer of pullulan coating, PET/PP/P + 1%CEO—polyethylene terephthalate/polypropylene film with a layer of pullulan coating and 1% clove essential oil, PET/PP/P + 5%CEO—polyethylene terephthalate/polypropylene film with a layer of pullulan coating and 5% clove essential oil, PET/PP/P + 10%CEO—polyethylene terephthalate/polypropylene film with a layer of pullulan coating and 10% clove essential oil.

The optical properties of packaging materials, including transparency and color, are critical factors for consumers since they allow visual inspection of the product’s freshness, quality, and overall appearance, reducing uncertainty before purchasing. Opacity is one of the parameters used to determine the optical transparency of food packaging materials. The results presented in Table 1 show that the PET/PP film exhibits an opacity value of 1.21, which indicates that it is a highly transparent material. Covering the PET/PP film with a layer of pullulan coating and pullulan coating with the addition of essential oil contributed to a gradual increase in the opacity (from 2.20 to 3.29). Luis et al. [46] also noted that incorporating rockrose essential oil into pullulan films reduced their transparency, which could be attributed to light scattering caused by the distribution of EO droplets within the biopolymer matrix. Nevertheless, Lei et al. [47] noted that transparent materials for food packaging are those with an opacity value below 5. The visual appearance of the coated PET/PP film shown in Figure 1 also confirms that the coating of the PET/PP film does not adversely affect the visibility of the text underneath the film, which confirms their good transparency properties.

The UV light transmittance at 280 nm and visible light transmittance at 600 nm for all films are presented in Table 1. The pullulan-coated PET/PP film was characterized by significantly lower UV light transmittance (measured at 280 nm) compared to an unmodified PET/PP film. After reinforcing the pullulan coating with clove essential oil at 1 and 5% concentration, no significant change in UV transmittance was observed compared to the PET/PP/P film. However, noticeable changes in the decreased UV light transmittance were recorded for the PET/PP film with a pullulan coating reinforced with 10% CEO. The UV light transmittance of the PET/PP/P + 10%CEO film was measured at 0.08%, indicating a reduction in UV transmission by 34% and 14% compared to the uncoated PET/PP film and the PET/PP film with a pullulan layer, respectively. Reducing UV light exposure helps preserve food quality by preventing photochemical reactions that cause color fading, off-flavor formation, and degradation of nutritional quality [48]. At the same time, visible light transmittance at 600 nm was also significantly lower through the PET/PP/P film (72.24%) compared to the PET/PP film (86.13%). In the films with lower clove oil concentration, i.e., PET/PP/P + 1%CEO and PET/PP/P + 5%CEO, no significant change in visible light transmittance was observed compared to the PET/PP/P film, whereas film with 10% clove oil (PET/PP/P + 10%CEO) significantly reduced light transmittance to 58.57%. Overall, the incorporation of clove essential oil into the pullulan coating, especially at higher concentrations, significantly improved the barrier properties of PET/PP films against UV and visible light. Moreover, the results highlight that PET/PP/P + 10%CEO film can be used as a packaging material for light-sensitive food products. Sharma et al. [49] also reported that the incorporation of clove oil into a composite film made of poly(lactide) and poly(butylene adipate-co-terephthalate) (PLA/PBAT–clove oil film) allowed obtaining packaging material with good UV protection and reduced visible light transmission. The enhanced UV-blocking properties were primarily attributed to the presence of phenolic compounds in clove essential oil that effectively absorb UV radiation [49], while the reduction in visible light transmission may be related to the light scattering caused by the presence of oil droplets dispersed in a polymer matrix [50].

In addition, the surface color of the tested films was analyzed by determining the parameters L*, a*, b* (Table 1). Covering the PET/PP film with a pullulan coating layer had no significant effect on color parameters (L*, a*, b*) compared to uncoated (pure) PET/PP films. However, slight color changes were observed for PET/PP films coated with pullulan-containing CEO. With the increasing concentration of CEO, the brightness of the films decreased slightly but significantly, as indicated by the values of the L* parameter. Also, the a* value decreased significantly while the b* value increased significantly with increasing CEO concentration in the pullulan coating. These results indicate that the incorporation of clove essential oil increased the shade intensity of the pullulan coating towards a yellow-green color, which is related to the yellow color of the clove essential oil used in the study. Similar results were observed by Sharma et al. [49], who found that the composite film made of a blend of poly(lactide)/poly(butylene adipate-co-terephthalate) with clove oil (PLA/PBAT–clove oil film) was characterized by a pale yellow color, which, as the authors suggest, was due to the presence of phenolic compounds that contributed to the yellowish hue of the composite film. However, visual inspection of the PET/PP films coated with pullulan and CEO did not reveal any noticeable greenish-yellow discoloration. This suggests that the differences observed in the a* and b* values between the PET/PP film and films with an active pullulan coating layer did not induce a perceptible color change, allowing the film to be classified as colorless. Additionally, based on the results of the total color difference, the ΔE for each tested PET/PP film with a pullulan coating did not exceed 2, which indicates a very small color difference compared to the unmodified PET/PP film. This suggests that experienced observers can detect the difference in color, whereas for most inexperienced observers (typically for most consumers) the difference in color will be unnoticeable [51].

### 2.2. Antibacterial and Antioxidant Activity of Films

The antibacterial efficacy of each PET/PP film composition was evaluated against four bacterial strains, including two Gram-negative (*E. coli* and *S.* Enteritidis) and two Gram-positive (*S. aureus* and *L. monocytogenes*) strains. Table 2 presents the number of bacteria recovered after 24 h of incubation on each film sample, the antimicrobial activity (R) quantified as a log reduction, and the percentage reduction in bacterial count determined for the PET/PP films with an active layer of pullulan coating. The results show that the PET/PP film with an active layer of pullulan coating containing CEO at a concentration of 1 to 10% showed antibacterial activity against all tested bacteria. However, some differences in the antibacterial effectiveness of films were observed, which were dependent on the concentration of CEO and bacterial strains. It was found that each PET/PP film with an active layer of pullulan coating showed more than 99.9% reduction in bacterial growth against both Gram-negative bacteria, which, according to the criteria, can be interpreted as a material with bactericidal activity (>3 log reduction). In turn, inhibition of the growth of Gram-positive bacteria was more dependent on the concentration of CEO in the pullulan coating. PET/PP/P +1%CEO showed moderate antimicrobial activity against *S. aureus* and *L. monocytogenes.* However, increasing the CEO content in the coating to 5% and 10% allowed us to obtain a material with strong antimicrobial activity. The observed difference in antimicrobial efficacy between Gram-negative (*E. coli*, *S.* Enteritidis) and Gram-positive bacteria (*S. aureus*, *L. monocytogenes*) can be attributed to the structural composition of their cell walls. Gram-negative bacteria have an outer membrane rich in lipopolysaccharides, which makes them more susceptible to the disruptive action of essential oils. In contrast, Gram-positive bacteria have a thicker peptidoglycan layer, which may provide increased resistance to antimicrobial agents.

**Figure 2:**
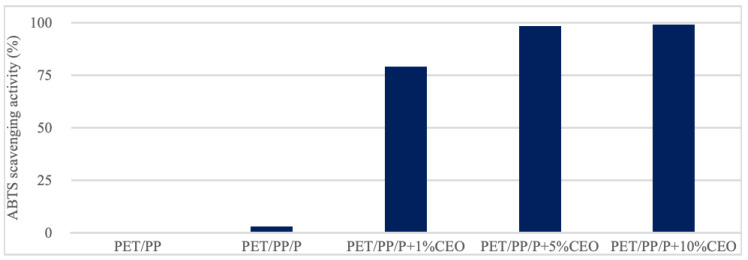
Antioxidant activity of unmodified and modified PET/PP films. PET/PP—polyethylene terephthalate/polypropylene film, PET/PP/P—polyethylene terephthalate/polypropylene film with a layer of pullulan coating, PET/PP/P + 1%CEO—polyethylene terephthalate/polypropylene film with a layer of pullulan coating and 1% clove essential oil, PET/PP/P + 5%CEO—polyethylene terephthalate/polypropylene film with a layer of pullulan coating and 5% clove essential oil, PET/PP/P + 10%CEO—polyethylene terephthalate/polypropylene film with a layer of pullulan coating and 10% clove essential oil.

To evaluate the antioxidant properties of the developed films, the ABTS radical scavenging activity assay was conducted, the results of which are presented in Figure 2. The uncoated PET/PP film and the PET/PP film with a pullulan coating did not show any antioxidant activity. In contrast, incorporating CEO into the pullulan coating significantly increased the antioxidant capacity. The PET/PP/P + 1%CEO, PET/PP/P + 5%CEO, and PET/PP/P + 10%CEO films exhibited an ABTS scavenging capacity of 78.9%, 98.2%, and 98.9%, respectively.

The antibacterial and antioxidant effect of PET/PP films with an active layer of pullulan coating is attributed to CEO, which is rich in bioactive compounds. The main constituent identified in CEO, obtained from buds or leaves, is eugenol, which mainly determines its biological activity, such as antioxidant, antimicrobial, antiviral, anti-inflammatory, and many other effects. Moreover, clove essential oil is approved by the U.S. Food and Drug Administration (FDA) and has GRAS (Generally Recognized as Safe) status [52]. In general, essential oils are recognized as a promising additive to the polymer material, imparting antimicrobial and antioxidant activity, thus enabling the development of active food packaging. However, their application also poses certain challenges, including high volatility and potential sensory impacts on food products. Consequently, these factors may restrict their use in food packaging [53]. Al-Hashimi et al. [54] reported that starch millet films fortified with clove essential oil exhibited excellent antioxidant potency adjusted to 85,96% when the film contained 3% oil. Additionally, the film displays an antibacterial effect against Gram-negative bacteria *(E. coli*, *P. aeruginosa*, *Enterobacter* sp.) and Gram-positive (*B. cereus* and *S. aureus*), with a slightly stronger effect against Gram-negative bacteria. Pullulan films containing caraway and oregano essential oils have been shown to have antibacterial and antifungal properties [34,55]. In other studies, rosemary, caraway, and fennel essential oil as active substances were introduced to ethylcellulose, which was used as a coating to cover the surface of a PLA film. The authors reported that the modified PLA film effectively inhibited the growth of *E. coli* and *S. aureus* [56].

### 2.3. Antimicrobial Activity of Model Active Package Bags

**Figure 3:**
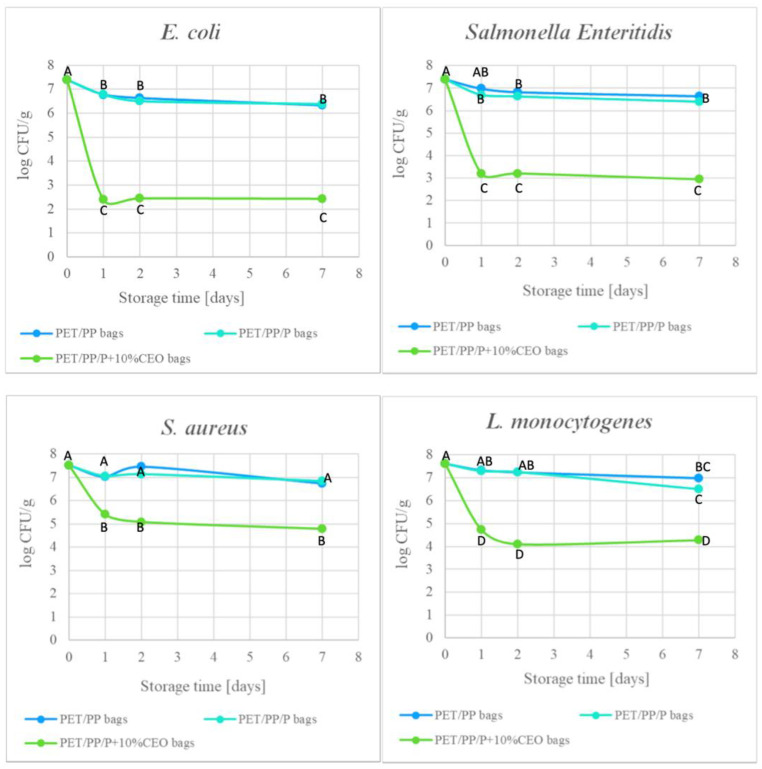
Inhibition of bacterial growth on spinach leaves packed in active packaging. A–D—different letters indicate statistical differences at *p* < 0.05, as determined by a two-way ANOVA followed by Tukey’s test; PET/PP bags—bags made of polyethylene terephthalate/polypropylene film, PET/PP/P bags—bags made of polyethylene terephthalate/polypropylene film with a layer of pullulan coating, PET/PP/P + 10%CEO—bags made of polyethylene terephthalate/polypropylene film with a layer of pullulan coating and 10% clove essential oil.

In this study, the effectiveness of the obtained active packaging was evaluated using spinach leaves artificially inoculated with foodborne pathogenic bacteria (Figure 3). Although all CEO-modified PET/PP films demonstrated antibacterial activity against the tested pathogens, PET/PP/P+10%CEO was selected as the model active packaging due to its significantly better optical properties. Its enhanced barrier to UV and visible light makes it suitable for preserving the quality of light-sensitive food products. 

The initial bacterial counts on spinach were 7.4, 7.4, 7.5, and 7.6 log CFU/g for *E. coli*, *S.* Enteritidis, *S. aureus*, and *L. monocytogenes*, respectively. During storage, bacterial counts on spinach leaves stored in PET/PP and PET/PP bags with a pullulan coating (PET/PP/P bags) decreased slightly but significantly within the range of 0.63–1.1 log CFU/g, likely due to the effect of low temperature of storage. Moreover, the change in the number of bacteria on spinach stored in PET/PP bags, which did not show antimicrobial activity, was found to be similar to that in PET/PP/P bags, indicating that these packages do not demonstrate antimicrobial properties. In contrast, the number of bacteria was effectively reduced on spinach stored in PET/PP bags with an active layer of pullulan coating containing 10% clove oil (PET/PP/P + 10%CEO bags). Initially, it was noted that active packaging exhibited greater inhibitory activity against Gram-negative bacteria than Gram-positive bacteria. After the first day of storing spinach in the active packaging, the bacterial counts of *E. coli* and *Salmonella* Enteritidis decreased by 5.0 and 4.21 log cycles, respectively. Thereafter, the population of both bacteria remained stable over the subsequent days of storage. Meanwhile, the bacterial count of *S. aureus* and *L. monocytogenes* after the first day of storage decreased by 2.1 and 2.9 log cycles, respectively. During further storage, bacterial count slightly decreased by approximately 0.5–0.6 log cycles. Overall, during the whole period of storage, PET/PP/P + 10%CEO demonstrated 3.7–5.0 and 2.0–2.8 log reduction in regard to PET/PP film for Gram-negative and Gram-positive bacteria, respectively. These results indicate that PET/PP/P + 10%CEO bags exhibited bactericidal activity against Gram-negative bacteria and a bacteriostatic effect against Gram-positive bacteria. Given that minimally processed vegetables are highly perishable, the application of this active packaging could provide a natural alternative to chemical preservatives, aligning with consumer demand for clean-label and eco-friendly solutions in the food industry. Devecioglu et al. [57] demonstrated that sachets made from poly(vinyl alcohol) (PVA) nanofiber film, enriched with clove oil encapsulated in cyclodextrin, effectively inhibited fungal growth when used for packaging bread slices.

### 2.4. Visual Appearance

**Figure 4:**
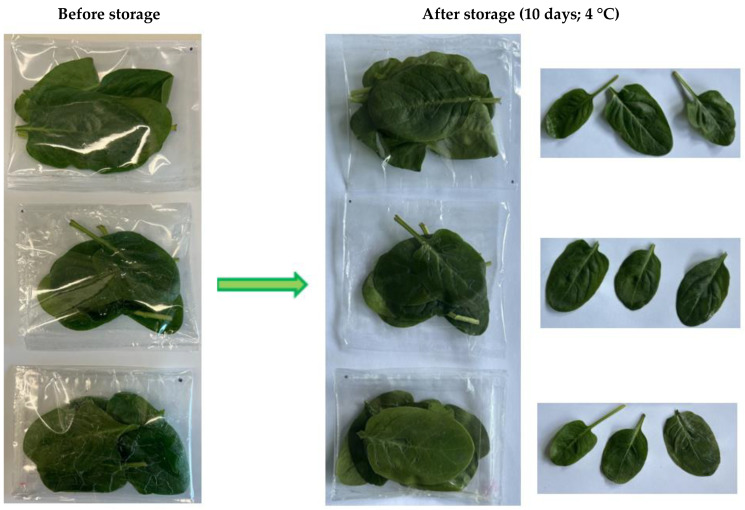
The visual appearance of spinach packed and stored in PET/PP bags, PET/PP/P bags, and PET/PP/P + 10%CEO bags. PET/PP bags—bags made of polyethylene terephthalate/polypropylene film, PET/PP/P bags—bags made of polyethylene terephthalate/polypropylene film with a layer of pullulan coating, PET/PP/P + 10%CEO—bags made of polyethylene terephthalate/polypropylene film with a layer of pullulan coating and 10% clove essential oil.

The visual attributes of perishable food products such as fruits and vegetables, including appearance, color, taste, smell, and texture, are key factors influencing consumer purchasing decisions [58]. Therefore, when evaluating active packaging, it is essential to assess not only its effectiveness in inhibiting microbial growth and preserving microbiological quality but also its impact on the visual characteristics of the product [59]. Figure 4 presents the visual appearance of spinach leaves stored in the active packaging bag model. Visual investigation indicated that the active packaging did not negatively affect the overall appearance of the spinach. Throughout the storage period, the spinach leaves retained their characteristic green color and the visually attractive qualities typical of fresh spinach. The lack of visible signs of deterioration, discoloration, damage, or decay indicates their good quality.

## 3. Materials and Methods

### 3.1. Materials

Laminated polyethylene terephthalate (PET)/polypropylene (PP) film (laminated PET/PP; 52 µm; water vapor permeability: 6 g/m^2^/24 h; oxygen vapor permeability: 95 cm^3^/m^2^/24 h) was used as the main packaging material, and pullulan (Hayashibara Co., Okayama, Japan) was used as a coating layer and carrier for clove essential oil (Vera-Nord, Legionowo, Poland), which serves as an antimicrobial substance. Tryptic soy broth (TSB) and Mueller-Hinton Agar (MHA) were purchased from BTL (Łódź, Poland). NaCl, glycerol, Tween 80, and potassium persulfate (K_2_S_2_O_8_) were from Chempur (Piekary Śląskie, Poland), PBS was obtained from VWR^®^ International (Solon, OH, USA) and ABTS was from Sigma Aldrich (Steinheim, Germany).

Bacterial strains: *Escherichia coli* ATCC 13067, *Salmonella enterica* subsp. *enterica* var. Enteritidis ATCC 13076, *Staphylococcus aureus* ATCC 25923, and *Listeria monocytogenes* ATCC 7644 were purchased from American Type Culture Collection and were stored in the a cryoprotective medium at −80 °C.

### 3.2. Preparation of Pullulan Film-Forming Solution

Prior to the final experiment, preliminary tests were conducted to optimize the formulation of the pullulan coating, ensuring uniform adhesion to PET/PP films. Based on this, the following pullulan coating solutions were prepared:

Control pullulan coating (P) was prepared by dissolving pullulan 10% (*w*/*v*) and glycerol 1% (*w*/*v*) in distilled water; the solution was mixed for 1 h using a magnetic stirrer (IKA, Warsaw, Poland).

Pullulan coating with 1% clove essential oil (P + 1%CEO) was prepared by dissolving pullulan (10% *w*/*v*), glycerol (1% *w*/*v*), and Tween 80 (1% *w*/*v*) in distilled water. The solution was mixed for 1 h using a magnetic stirrer (IKA, Poland). Subsequently, clove essential oil (1% *w*/*v*) was added, and the mixtures were homogenized by ultrasonication at 20 kHz and 60% amplitude for 5 min. To maintain the temperature below 30 °C, the samples were placed in an ice bath during ultrasonication.

Pullulan coating with 5% clove essential oil (P + 5%CEO) was prepared by dissolving pullulan (10% *w*/*v*), glycerol (1% *w*/*v*), and Tween 80 (3% *w*/*v*) in distilled water. The solution was mixed for 1 h using a magnetic stirrer (IKA, Poland). Subsequently, clove essential oil (5% *w*/*v*) was added, and the mixtures were homogenized by ultrasonication at 20 kHz and 60% amplitude for 5 min. To maintain the temperature below 30 °C, the samples were placed in an ice bath during ultrasonication.

Pullulan coatings with 10% clove essential oil (P + 10%CEO) were prepared by dissolving pullulan (10% *w*/*v*), glycerol (1% *w*/*v*), and Tween 80 (6% *w*/*v*) in distilled water. The solution was mixed for 1 h using a magnetic stirrer (IKA, Poland). Subsequently, clove essential oil (10% *w*/*v*) was added, and the mixtures were homogenized by ultrasonication at 20 kHz and 60% amplitude for 5 min. To maintain the temperature below 30 °C, the samples were placed in an ice bath during ultrasonication.

### 3.3. Application of Pullulan Film-Forming Solution onto PET/PP Films

Pullulan film-forming solution (with and without clove essential oil) was then applied to sheets of a PET/PP film using the automatic film applicator Byko-Drive V (BYK-Gardner, Geretsried, Germany) and a frame applicator with a gap depth of 150 um. The application rate of the film-forming solution was 20 mm/s. The PET/PP film with a pullulan coating was left to dry in the laminar chamber (previously sterilized by UV radiation) at room temperature for 24 h.

Finally, the following modifications of the PET/PP film were prepared for this study: the PET/PP film with an active layer of pullulan coating with 1% clove essential oil (PET/PP/P + 1%CEO), the PET/PP film with an active layer of pullulan coating with 5% clove essential oil (PET/PP/P + 5%CEO), and the PET/PP film with an active layer of pullulan coating with 10% clove essential oil (PET/PP/P + 10%CEO). Additionally, a non-modified/uncoated PET/PP film and a PET/PP film with a layer of pullulan coating (without clove essential oil; PET/PP/P) were prepared and served as a control.

**Figure 5:**
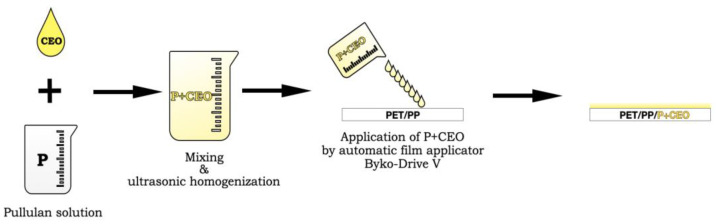
Preparation and application of the pullulan film-forming solution with clove essential oil (CEO) onto PET/PP films.

The scheme of the preparation and application of pullulan film-forming solution with clove essential oil (CEO) onto PET/PP are presented in Figure 5.

### 3.4. Physical Characteristics of Film

#### 3.4.1. Thickness

The thickness of the unmodified and modified PET/PP film was measured using a thickness gauge (BYKO-Test 4500, BYK-Gardner, Geretsried, Germany). Measurements were performed in ten repetitions at randomly selected locations across different areas of each film sample, specifically at the center and near the edges.

#### 3.4.2. Optical Properties (Opacity and Light Transmittance)

The opacity and light transmittance of unmodified and modified PET/PP films were measured using a Metertech UV–VIS SP-8001 spectrophotometer (Metertech Inc., Taipei, Taiwan). Film samples were cut into 1 × 5 cm pieces and inserted directly into the spectrophotometer’s test chamber. The absorbance of the films was measured at a wavelength of 600 nm. The experiment was conducted with three replications. The opacity of the films was calculated using Equation (1) [60]:

(1) $$Opacity=\frac{{Abs}_{600}}{t}$$

where *Abs*_600_ is the absorbance value at 600 nm and *t* is the film thickness (mm).

Additionally, the UV barrier and visible light transmission properties of the films were evaluated by measuring transmittance at 280 nm and 600 nm, respectively. The results were present as a percentage of light transmittance.

#### 3.4.3. Color Analysis

The color parameters (L*, a*, b*) of unmodified and modified PET/PP films were determined using a CR-400 colorimeter (Minolta, Tokyo, Japan). Measurements were performed in ten repetitions at randomly selected locations on each film sample. Additionally, the total color difference (ΔE) was calculated using Equation (2):

(2) $$\Delta E=\sqrt{{\left(L_{m}-L_{c}\right)}^{2}+{\left(a_{m}-a_{c}\right)}^{2}+{\left(b_{m}-b_{c}\right)}^{2}}$$

where *L_c_*, *a_c_*, and *b_c_* are values for uncoated/unmodified PET/PP films, and *L_m_*, *a_m_*, and *b_m_* are values for modified PET/PP films.

### 3.5. Antibacterial Activity of Films

The frozen stock culture of each tested strain of bacteria was activated on a TSB medium at 37 °C for 24 h. Then, bacteria were transferred and spread onto Petri dishes with TSB medium and cultured at 37 °C for 24 h. Afterward, bacterial cells were harvested and transferred to PBS to obtain an optical density of 0.5° on the McFarland scale measured using a Densimat instrument (bioMérieux, Marcy-l’Étoile, France), corresponding to the cell concentration in the suspension of 1–2 × 10^8^ CFU/mL. The prepared bacterial solution served as the final bacterial inoculum.

Measurement of antibacterial activity of unmodified and modified PET/PP films was determined according to the protocol based on ISO 22196:2011 [61] with slight modifications. Briefly, films were cut into 3 × 3 cm pieces and placed in sterile 90 mm Petri dishes. Hundred µL of the final bacteria inoculum (approx. 1– 2 × 10^8^ CFU/mL) were pipetted onto films as 10-μL droplets, evenly distributed over the entire surface of the film. Samples were incubated at 37 °C for 24 h. To determine the recovery of bacteria from the film surface, 10 mL of PBS was poured into each Petri dish containing a test piece of film, and then the plates were mixed thoroughly to wash away the bacteria. Next, appropriate decimal dilutions were prepared, spread onto MHA plates, and incubated at 37°C for 24 h. After incubation, the colonies on each plate were counted and the number of bacteria was calculated using Equation (3):

(3) $$N=\frac{100\times C\times V\times D}{A}$$

where C is the average of CFU count on plates; D is the dilution factor; V is the volume of PBS (mL); A is the surface of the film (mm^2^).

The antibacterial activity (R) was determined using Equation (4):

R = Ut − At(4)

where R is the antibacterial activity; Ut is the average of the log CFU/cm^2^, recovered from unmodified/uncoated PET/PP film after 24 h.

At is the average of the log CFU/cm^2^, recovered from PET/PP film with an active pullulan layer after 24 h.

The percentage of bacterial reduction was calculated using Equation (5):

(5) $$\mathrm{\% of growth reduction}=\frac{NB\_control-NB\_sample}{NB\_control}\times 100\%$$

NB_control is the number of bacteria colonies (CFU/cm^2^) recovered from the unmodified/uncoated PET/PP film after 24 h.

NB_sample is the number of bacteria colonies (CFU/cm^2^) recovered from the PET/PP film with an active pullulan layer after 24 h.

The antimicrobial activity (R) of the PET/PP film with an active pullulan coating layer was evaluated based on the following classification: no antimicrobial activity (≤0.5 log microbial growth reduction, <68.4% reduction); slight antimicrobial activity (0.5–1 log reduction, from 68.4% to < 90% reduction); moderate antimicrobial activity (>1 to ≤2 log reduction, 90% to <99% reduction); good antimicrobial activity (2 to <3 log reduction, from 99% to <99.9% reduction); and very good antimicrobial activity (>3 log reduction, >99.9% reduction) [61]. Additionally, percentage bacterial reduction was used to determine whether the composite film exhibited bactericidal activity (>99.9% reduction in bacterial counts) or bacteriostatic activity (from 90% to 99.9% reduction in bacterial counts) [62].

### 3.6. ABTS Radical Scavenging Activity of Films

The antioxidant activity of each PET/PP film was evaluated by the ABTS radical scavenging method according to the procedure reported by Ma et al. [63], with slight modification. A mixture of ABTS and potassium persulfate at concentrations of 7.00 mmol/L and 2.45 mmol/L, respectively, was prepared and left at room temperature in the dark for 16 h before being used to generate ABTS radical cations (ABTS^•+^). Then, the obtained ABTS^•+^ solution was diluted in PBS to an absorbance of 0.70 ± 0.05 at λ = 734 nm (Metertech UV–VIS SP-8001). Each PET/PP film (5 mg) was immersed in 4 mL of ABTS solution and kept for 3 min in the dark at room temperature, and after that, the absorbance at 734 nm was measured. The experiment was conducted with three replications. The antioxidant activity for each PET/PP film was calculated using Equation (6):

(6) $$\mathrm{ABTS scavenging activity}\;(\%)=\frac{Abs\_control-Abs\_sample}{Abs\_control}\times 100\%$$

where Abs_control is the absorbance value of blank ABTS solution and Abs sample is the absorbance value of ABTS solution containing test film.

### 3.7. Preparation of a Model Packaging Bags

In the first step, polypropylene (PET/PP) films with an active layer of pullulan coating containing 10% clove oil (PET/PP/P + 10%CEO) and a layer of pullulan coating without clove essential oil (PET/PP/P) were prepared as described above. Additionally, uncoated/unmodified PET/PP films were also prepared. Then, the coated and uncoated PET/PP film sheets were cut into 7 × 10 cm rectangles. These rectangles were subsequently sealed along their edges using an impulse welder (PFS/FS 300 C, ITAX, Bydgoszcz, Poland) to obtain model packaging bags.

#### 3.7.1. Preparation and Inoculation of Spinach Leaves

Fresh spinach leaves purchased from the local market in Warsaw, Poland, were transported to the laboratory in original producer bags and stored in the refrigerator at 4 °C until the laboratory research process began. For the study, whole and undamaged leaves were selected, which were then washed twice in sterile water and placed inside the laminar chamber for 1 h to dry. In the meantime, while keeping them in the chamber, spinach leaf surfaces (each side) were exposed to UV radiation for 10 min to eliminate native microflora. After that, spinach was artificially infected with testing pathogenic bacteria, including *E. coli*, *S.* Enteritidis, *S. aureus*, and *L. monocytogenes*. Spinach samples were spot-inoculated with 200 µL of final bacterial inoculum (approximately 1–2 × 10^8^ CFU/mL; prepared as above) and left to dry for 30 min to allow bacterial cells to adhere to the spinach leaf surfaces. The experiments were performed separately for each bacterial strain tested. The initial bacterial concentration on spinach leaves after inoculation was 7.4, 7.4, 7.5, and 7.6 log CFU/g for *E. coli*, *S.* Enteritidis, *S. aureus*, and *L. monocytogenes*, respectively.

#### 3.7.2. Packaging and Microbial Enumeration

Inoculated spinach leaves (5 g) were packed into model packaging bags: PET/PP bags, PET/PP/P bags and PET/PP/P + 10%CEO bags. The packed spinach leaves were stored at 4 °C for 7 days. On days 1, 2, and 7, samples of spinach leaves (1 g) were removed from uncoated and coated model bags and transferred to a stomacher bag with 9 mL of 0.85% sodium chloride solution. After manual mixing, a series of decimal dilutions were made, and 1 mL of the appropriate dilutions was poured onto MHA plates, which were incubated at 37 °C for 24 h, and bacterial colonies were counted using an automatic colony counter (ProtoCol3, Synbiosis, Cambridge, UK). The results were expressed as log CFU/g. The experiments were performed with three replications per condition.

### 3.8. Statistical Analysis

Statistical analysis was performed using Statistica 13.3 PL (TIBCO, Palo Alto, CA, USA). A one-way ANOVA, followed by Tukey’s test for multiple comparisons (*p* < 0.05), was conducted to evaluate the physical characteristics of the films. A two-way ANOVA, followed by Tukey’s test (*p* < 0.05), was applied to assess the antimicrobial activity of the model active packaging bags.

## 4. Conclusions

In this study, an active packaging material based on a polypropylene (PET/PP) film modified with a pullulan coating enriched with clove essential oil was successfully developed. Covering the PET/PP film with a layer of pullulan coating and pullulan coating with the addition of clove essential oil contributed to a gradual increase in thickness and opacity. Nevertheless, all modified PET/PP films were still considered a transparent material. Moreover, the incorporation of clove essential oil into the pullulan coating, particularly at higher concentrations, enhanced the UV and visible light barrier properties of the PET/PP films while not causing a noticeable color change, allowing the films to be classified as colorless. Additionally, the PET/PP film with an active layer of pullulan-containing CEO exhibits good antioxidant and antibacterial activity against Gram-negative (*E. coli*, *S*. Enteritidis) and Gram-positive bacteria (*S. aureus*, *L. monocytogenes*). The application of active packaging effectively reduces the number of pathogenic bacteria on spinach leaves. It was noted that active packaging exhibited greater inhibitory activity against Gram-negative bacteria than Gram-positive bacteria. In addition, no adverse effect on the visual qualities of spinach samples packed and stored in active material was observed. These findings underscore the practical viability of this active packaging for the fresh produce industry, providing a natural and effective alternative to chemical preservatives. Nevertheless, to comprehensively evaluate the potential of the PET/PP films modified with pullulan-containing CEO, it is crucial to analyze their mechanical and barrier properties, as these factors play an important role in evaluating their suitability as packaging materials.

For future research, it is recommended to conduct a sensory analysis of such active packaging to assess its impact on the odor or taste of fresh food products. Additionally, the mechanical and barrier properties of the modified PET/PP film, which may influence its practical application in packaging, should be evaluated in subsequent studies.

## Figures and Tables

**Table 1:** Optical and color properties of unmodified and modified PET/PP films.

Film Sample	Thickness [mm]	Opacity [a.u./mm]	Transmittance (%)		Color			
			280 nm	600 nm	L*	a*	b*	ΔE
PET/PP	0.052 ± 0.00 a **	1.21 ± 0.04 a	0.121 ± 0.003 c	86.14 ± 0.46 c	91.61 ± 0.65 c	−0.46 ± 0.03 a	0.53 ± 0.05 a	-
PET/PP/P	0.064 ± 0.005 b	2.20 ± 0.08 b	0.093 ± 0.005 b	72.25 ± 1.42 b	91.18 ± 068 b	−0.66 ± 0.15 bc	0.63 ± 0.05 b	0.49
PET/PP/P + 1%CEO	0.065 ± 0.004 b	2.30 ± 0.13 c	0.095 ± 0.005 b	70.93 ± 1.63 b	90.62 ± 0.35 a	−0.64 ± 0.18 b	0.64 ± 0.08 b	1.02
PET/PP/P + 5%CEO	0.069 ± 0.006 c	2.61 ± 0.28 d	0.091 ± 0.009 b	68.2 ± 05.76 b	90.56 ± 0.20 a	−0.74 ± 0.08 cd	0.93 ± 0.19 c	1.16
PET/PP/P + 10%CEO	0.073 ± 0.007 d	3.29 ± 0.38 e	0.080 ± 0.007 a	58.57 ± 5.41 a	90.75 ± 0.51 a	−0.78 ± 0.11 d	1.12 ± 0.15 d	1.10

** a–e—different letters in the column indicate statistical differences at *p* < 0.05. PET/PP—unmodified film, PET/PP—polyethylene terephthalate/polypropylene film, PET/PP/P—polyethylene terephthalate/polypropylene film with a layer of pullulan coating, PET/PP/P + 1%CEO—polyethylene terephthalate/polypropylene film with a layer of pullulan coating and 1% clove essential oil, PET/PP/P + 5%CEO—polyethylene terephthalate/polypropylene film with a layer of pullulan coating and 5% clove essential oil, PET/PP/P + 10%CEO—polyethylene terephthalate/polypropylene film with a layer of pullulan coating and 10% clove essential oil.

**Table 2:** Antimicrobial activity of unmodified and modified PET/PP films.

Bacteria Strains	Film Sample	Number of Bacteria (log CFU/cm^2^)	R (Log Reduction)	% Reduction
*E. coli*	PET/PP	4.03	-	
	PET/PP/P	3.83	0.14	
	PET/PP/P + 1% CEO	n/d *	4.03	>99.9%
	PET/PP/P + 5% CEO	n/d	4.03	>99.9%
	PET/PP/P + 10% CEO	n/d	4.03	>99.9%
*Salmonella* Enteritidis	PET/PP	3.72	-	-
	PET/PP/P	3.91	0	
	PET/PP/P + 1% CEO	n/d	3.72	>99.9%
	PET/PP/P + 5% CEO	n/d	3.72	>99.9%
	PET/PP/P + 10% CEO	n/d	3.72	>99.9%
*S. aureus*	PET/PP	5.55	-	
	PET/PP/P	5.80	0	
	PET/PP/P + 1% CEO	3.77	1.78	98.95%
	PET/PP/P + 5% CEO	n/d	5.55	>99.9%
	PET/PP/P + 10% CEO	n/d	5.55	>99.9%
*L. monocytogenes*	PET/PP	3.34	-	
	PET/PP/P	3.26	0.08	
	PET/PP/P + 1% CEO	2.17	1.17	93.2%
	PET/PP/P + 5% CEO	n/d	3.34	>99.9%
	PET/PP/P + 10% CEO	n/d	3.34	>99.9%

* n/d—not detected. PET/PP—polyethylene terephthalate/polypropylene film, PET/PP/P—polyethylene terephthalate/polypropylene film with a layer of pullulan coating, PET/PP/P + 1%CEO—polyethylene terephthalate/polypropylene film with a layer of pullulan coating and 1% clove essential oil, PET/PP/P + 5%CEO—polyethylene terephthalate/polypropylene film with a layer of pullulan coating and 5% clove essential oil, PET/PP/P + 10%CEO—polyethylene terephthalate/polypropylene film with a layer of pullulan coating and 10% clove essential oil.

## Data Availability

Data are available in the article.
